# A SYSTEMATIC REVIEW OF COOPERATIVE EXTENSION COMMUNITY-BASED RESEARCH FOR LATE LIFE MENTAL HEALTH

**DOI:** 10.1093/geroni/igae098.1698

**Published:** 2024-12-31

**Authors:** Naomi Meinertz, Mary Dozier, Kate Perepezko, JeongEun Lee

**Affiliations:** University of Missouri, Columbia, Missouri, United States; Mississippi State University, Mississippi State, Mississippi, United States; Scripps Gerontology Center, Lansdale, Pennsylvania, United States; Iowa State University, Ames, Iowa, United States

## Abstract

Rural-dwelling older adults have greater mental health disparities with higher rates of depression and anxiety compared to their urban counterparts; however, reaching these communities to understand and address the specific psychological needs of rural-dwelling older adults remains a barrier for gerontologists. Due to the extensive reach of the Cooperative Extension system (Extension), with representation in every county across the U.S., Extension is primed to facilitate connections between researchers and rural communities to improve mental health outcomes for older adults. Despite the benefits of such partnerships, Extension is largely unknown and underutilized by gerontological researchers, which leaves many older adults unconnected with resources that could improve their mental health outcomes. To better understand this gap, we conducted a systematic review of gerontological studies that utilized Extension to reach and positively impact rural older adult mental health. Using the methods and results from these studies, we highlight the principles of community-based participatory research (CBPR) that make this gerontological research possible and beneficial for older adult mental health outcomes. We also explore the characteristics important for these collaborative relationships and underscores the role that Extension plays in improving mental health outcomes of older adults. Our review will contribute to gerontological research by outlining the advantages of CBPR conducted in partnership with the Extension system and providing guidance for researchers to make these connections that will reduce the mental health disparities between rural and urban older adults.

